# Challenges and Factors Affecting Child, Adolescents, Young Adults, and Their Parents in Returning to School After Remote Learning in COVID Pandemic

**DOI:** 10.1192/j.eurpsy.2023.875

**Published:** 2023-07-19

**Authors:** S. Ashraf, S. Srinivas, A. Bachu, R. Asim, F. Kim, D. Simpson, K. Shah

**Affiliations:** 1 Northpointe Psychiatry, Lewisville, TX, United States; 2 Psychiatry, A.J.Institute of Medical Sciences and Research Center, Mangaluru, Karnataka, India; 3 UAMS- Baptist Health, North Little Rock, United States; 4 Fatima Jinnah Medical University, Lahore, Pakistan; 5 Psychiatry; 6 Oklahoma State University/Griffin Memorial Hospital Psychiatry Residency Program, Norman, OK; 7 Wake Forest University, Winston-Salem, NC, United States

## Abstract

**Introduction:**

The COVID pandemic caused an unprecedented public health crisis and adversely impacted children’s well-being. It has negatively affected children’s mental health due to social isolation, human losses, and remote learning. Our goal is to learn about the challenges and factors that these children and young adults face upon returning to school and college, which could further decline their mental health. We also need to understand parents’ concerns about this transition to a back-to-school routine.

**Objectives:**

1) To learn about the mental health challenges for children, adolescents, and young adults returning to school after the beginning of the COVID pandemic.

2) To identify the factors and challenges that parents and caregivers face during the COVID regarding the return of their children to school.

**Methods:**

We conducted a literature search using relevant medical subject heading (MeSH) terms in PubMed, PubMed Central, Web of Science, and Medline databases. We identified all published relevant articles until June 4, 2021. After a thorough review of relevant published articles until October 30, 2022, we included 5 articles in our qualitative synthesis.

**Results:**

A cross-sectional study in China measured depression, anxiety, and social support in back-to-school students via PHQ-9, GAD-7, and SSQ, respectively. They found a significant rise in anxiety and depression among these students. This correlation was weak at higher social support. Data collected from 15 children’s hospitals found that students want to participate actively in returning to school and the recovery process as they are concerned about their future, family, and society. Another 2021 cross-sectional study in Texas revealed that parents are concerned about their children’s health and prefer an onsite-virtual hybrid learning setup over in-person learning (Limbers C. A. et al. *The Journal of school health* 2021; *91*(1), 3–8.). Parents in Italy favored school reopening with reduced student numbers (70.1%), social distancing within classes (45.3%), and masks as they were concerned about their children due to COVID (Pierantoni, L et al. 2021; *Acta paediatrica (Oslo, Norway : 1992)*, *110*(3), 942–943 ). Fewer White parents were supportive of a mask mandate for students and staff members (62.5%) than parents of ethnicities like Hispanic (79.5%, p= 0.026) and other racial/ethnic groups (66.9%, p = 0.041) (Gilbert, L. K. et al. *MMWR. Morbidity and mortality weekly report 2020; 69*(49), 1848–1852).

**Conclusions:**

The return to school after COVID is challenging for students and parents due to the rise in anxiety and depression in children. Social support has been found to be protective of children’s mental health. Future well-designed studies should identify challenges and factors that can help safeguard children’s mental health and develop appropriate policies.

**Disclosure of Interest:**

None Declared

